# Experimental analysis of control technology and deformation failure mechanism of inclined coal seam roadway using non-contact DIC technique

**DOI:** 10.1038/s41598-021-00462-9

**Published:** 2021-10-22

**Authors:** Xianyu Xiong, Jun Dai, Yibo Ouyang, Pan Shen

**Affiliations:** 1 School of Architecture and Civil Engineering, Xi’an University of Science and Technology, Xi’an, 710054 Shaanxi China; 2 College of Energy Science and Engineering, Xi’an University of Science and Technology, Xi’an, 710054 Shaanxi China

**Keywords:** Energy science and technology, Engineering

## Abstract

The deformation and failure forms of inclined coal seam roadway under the joint action of dip angle and various geological conditions are complex, and there is a lack of targeted support measures, which brings great problems to the stability control of roadway surrounding rock. In order to safely and economically mine inclined coal seams, taking the engineering geology of Shitanjing No. 2 mining area as the background, and the physical similarity model of right-angle trapezoidal roadway in inclined coal seam, in which the non-contact digital image correlation (DIC) technology and the stress sensor is employed to provide full-field displacement and stress measurements. The deformation control technology of the roadway surrounding rock was proposed, verified by numerical simulation and applied to engineering practice. The research results show that the stress and deformation failure of surrounding rock in low sidewall of roadway are greater than those in high sidewall, showing asymmetric characteristics, and the maximum stress concentration coefficients of roadway sidewall, roof and floor are 4.1, 3.4 and 2.8, respectively. A concept of roadway "cyclic failure" mechanism is proposed that is, the cyclic interaction of the two sidewalls, the sharp angles and roof aggravated the failure of roadway, resulting in the overall instability of roadway. The roadway sidewall is serious rib spalling, the roof is asymmetric "Beret" type caving arch failure, and the floor is slightly bulging. On this basis, the principle of roadway deformation control is revealed and asymmetric support design is adopted, and the deformation of roadway is controlled, which support scheme is effective.

## Introduction

The coal resources in the western region account for about 86.5% of China's coal reserves, of which the inclined coal seam accounts for about 35% of its reserves, and there are high-quality coal seams with high mining value^1–4^. Due to the influence of dip angle, section type of roadway and geological conditions, the mining of inclined coal seam is different from that of the general coal seam and has its particularity. Especially after the excavation of the coal seam roadway, the deformation and failure of surrounding rock show asymmetric characteristics, and the roadway often occurs rib spalling, roof fall accidents^5–10^. Therefore, it is of great significance to study the deformation and failure mechanism of roadway in inclined coal seam to effectively support roadway to ensure its stability.

At present, many scholars have studied the deformation control of roadway surrounding rock in inclined coal seam by using the physical model test, theoretical analysis and numerical simulation. Manchao He et al. studied the stability of surrounding rock during the excavation of rectangular roadway in inclined coal seam through physical model test, and obtained that the excavation failure area presents the form of disturbance zone parallel to the rock stratum, and the failure mechanism is mainly bedding fracture^11,12^. Shuai Zhang et al. analyzed the stress distribution and deformation characteristics of coal pillar and roadway support gob-side entry in deep inclined coal seam through theoretical analysis and numerical simulation, and concluded that 5 m wide coal pillar can ensure that the coal pillar is at a low stress level, the deformation of roadway surrounding rock is within a reasonable range, and the roadway support is in good condition^13^. Weitao Liu et al. studied the stress distribution of roadway surrounding rock under mining stress in inclined coal seam floor by using the semi infinite body theory in elasticity, and calculated the horizontal stress and shear stress at any point in the roadway floor^14,15^. Hai Wu established a mechanical model according to the mechanics of materials, analyzed the stress distribution and deformation failure characteristics of vertical wall arch roadway in the deep inclined coal seam, and put forward the control technology asymmetric deformation of the roadway^16^. Jianjun Zhao et al. conducted a physical model test on the deformation and failure process of overlying strata in the goaf roof of the gently inclined coal seam, and concluded that the tensile stress concentration occurred at the boundary of the goaf after the coal seam mining, bending deformation dominated by settlement occurred, and finally sliding failure^17^. Xiaoming Sun et al. used FLAC3D numerical simulation software to study the mechanism of asymmetric deformation of vertical wall arch roadway in deep inclined coal seam, and proposed asymmetric coupling support scheme, and strengthened support in key parts^18^. Xinzhong Chen established a numerical model of gob-side entry driving in deep inclined coal seam, and analyzed the asymmetric large deformation characteristics of surrounding rock of this kind of roadway, the deformation of narrow coal pillar wall and floor is much greater than that of solid coal wall and roof, and the overall section convergence rate of roadway is large^19^. Hongyun Yang et al. carried out numerical simulation analysis on soft roof failure mechanism of mining roadway in gently inclined coal seam and optimized the support scheme^20^. Wei Zhang et al. established the mechanical structure model, analyzed the surrounding rock deformation mechanism of right angle trapezoidal roadway in deep gently inclined three-soft coal seam, and optimized the supporting scheme^21^.

At present, the research on the control of surrounding rock deformation of inclined coal seam roadway mainly focuses on the rectangular and straight wall arch roadway and mostly studies the independent parts of the roadway. There are few systematic studies on the roof's coupling failure mechanism, two sidewalls and floor of the inclined coal seam rectangular trapezoidal roadway. In this paper, the deformation and failure mechanism of right-angle trapezoidal roadway in inclined coal seam under the condition of no support is investigated by physical model tests of large-scale variable angle, in which the stress sensor and the non-contact Digital Image Correlation (DIC) technique is employed to provide stress and full-field displacement measurements, respectively. For deformation and failure characteristics, an asymmetric support plan is proposed to strengthen key parts' support, which is verified by numerical simulation and applied to engineering practice.

## Engineering background

**Figure 1:**
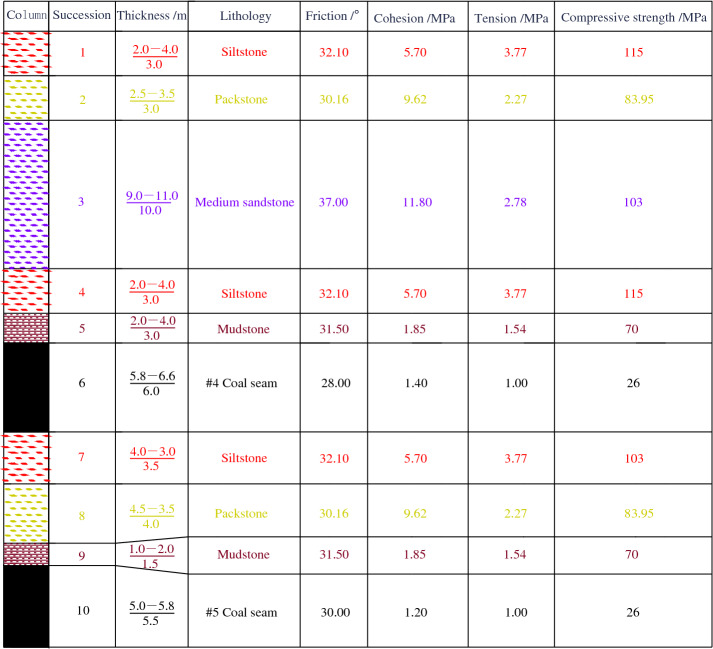
Lithology histogram.

The No. 2 Mining Area of Shitanjing is located in the high mountain area of western Ningxia. The dip angle of coal strata is 18°–27°, with an average of 23°. The mining coal seam is the #4 layer of coal. The buried depth is about 405.6–480.1 m, the thickness is 5.8–6.6 m, with an average of 6 m. The roof is mudstone, and the floor is siltstone. It is a typically inclined coal seam. The lithology histogram is shown in Fig. 1, it shows the material data obtained from the field drilling measurement in Shitanjing No. 2 mining area. The roadway section is designed as a right-angle trapezoid with a width of 4.5 m and the low sidewall height of 3 m.

**Figure 2:**
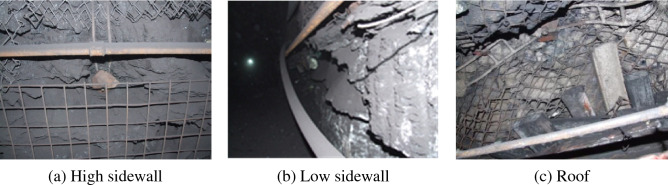
Deformation and destruction of roadway site.

Under the combined support of anchor bolt, net and cable scheme, the right angle trapezoid roadway in inclined coal seam displays unsymmetrical deformation characteristics, as shown in Fig. 2. Serious rib spalling undergoes in the two sidewalls of roadway, and the deformation of the low sidewall of roadway is greater than that of the high sidewall, and the roof undergoes intense sidestepped subsidence. The metal mesh is torn in some places, the bolt plates are sunken and have loosened, and both the anchor bolts and anchor cables display considerable bending deformation, indicating that the original supporting effect is low.

## Physical model test

### Test setup

**Figure 3:**
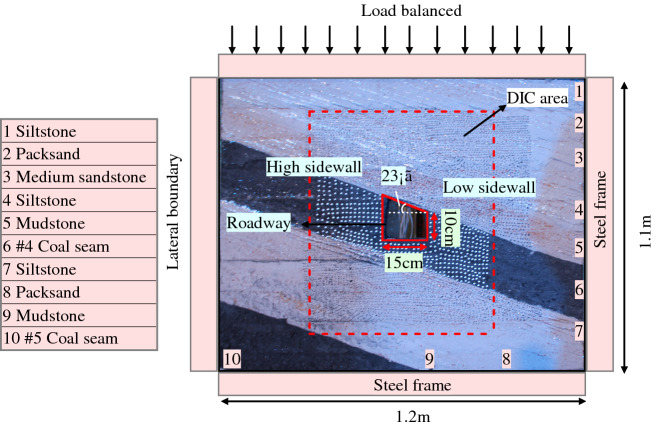
Physical simulation test system.

In order to control the deformation of asymmetric roadway surrounding rock in inclined coal seam and mine inclined coal seam safely and efficiently, a large-scale variable angle model test (length × width × height = 1.2 m × 0.12 m × 1.1 m) apparatus is assembled to reveal the deformation failure mechanism of roadway surrounding rock without support (see Fig. 3). In this study, in order to simulate the effect of in-situ stress, uniform load was applied on the top of the model to trigger the failure of surrounding rock after excavation (Jia and Tang 2008; Lee et al. 2010). Simplifications adopted in the model tests are given as follows: (1) The cross-section of right angle trapezoidal roadway in the inclined coal seam with the dip angle of 23° in Shitanjing No.2 Mining Area is used; (2) No support system is considered, in order to reveal the source of roadway deformation and failure; (3) The influence of roadway excavation process is not considered.

Two hydraulic jacks are used to apply uniform load on the top surface of the physical model. Starting from 0.0385 MPa, the staged loading method is performed, as shown in Table 1.

**Table 1:** Loading scheme.

Load times	1	2	3	4	5	6	7	8	9	10	11	12	13
Load (kN)	3	4	5	6	7	8	9	10	11	12	13	14	15
Load (MPa)	0.021	0.028	0.035	0.042	0.049	0.063	0.07	0.077	0.084	0.091	0.098	0.105	0.112

The real profile of right angle trapezoidal roadway in inclined coal seam is adopted in the model test. Based on the laws of similitude^22–26^, the similarity constants of the geometry, bulk density, and stress of the calculated model are determined to be 30, 1.6, and 48 respectively by Eq. (1), then the width of the roadway and the height of the low sidewall of roadway are 150 mm and 100 mm respectively. Meet the requirements of the roadway to boundary distance/roadway radius ≥ 3^27,28^. The model's prototype dimensions are 33 m in height, 36 m in length and 3.6 m in width.

1 $$ \alpha_{\sigma } = \frac{{r_{P} }}{{r_{M} }}\alpha_{L} = \alpha_{r} .\alpha_{L} $$

where *α*_*σ*_ is the strength and stress similarity constant; *α*_*r*_ is the similarity constant of bulk density; *α*_*L*_ is the geometric size similarity constant; *r*_*P*_ is the average bulk density of the original rock, taking 2.5 g/cm^3^; The *r*_*M*_ is the bulk density of the model material, generally *r*_*M*_ in 1.5–2.5 g/cm^3^ is suitable, too large molding compaction is difficult, too small makes the model material loose and difficult to form.

Simulated materials with a mixture of sand, gypsum and CaCO_3_ are used to mimic the physical and mechanical properties of coal and rock strata, and mica flake is used to simulate the joint layer between coal and rock. In this study, the proportion of similar materials is optimized through the orthogonal test. The optimized proportion can make the model materials accurately achieve the expected mechanical strength. The proportion of similar materials is shown in Table 2. The meaning of proportioning number: the first digit represents the ratio of sand to binder, and the second and third numbers represent the proportion of gypsum and CaCO_3_ in the two cements, such as 737 in the table, which means the sand binder ratio is 7:1, and the ratio of gypsum and CaCO_3_ in a cement is 3:7.

**Table 2:** Ratio of physical similar material simulation test.

Layer number	Kinds of strata		Layer thickness (cm)	Ratio (sand: gypsum: CaCO_3_: coal)
1	Siltstone		10	737
2	Packsand		10	837
3	Medium sandstone		33	728
4	Siltstone		10	737
5	Mudstone		10	828
6	#4 Coal seam		20	21:1:2:21
7	Siltstone		12	737
8	Packsand		13	837
9	Mudstone		5	828
10	5 #Coal seam		18	21:1:2:21

### Instrumentation

The monitoring system consists of miniature earth pressure sensor (L–YB–150 (φ28 mm × 10 mm), AD–64 data acquisition instrument, DIC, displacement meter and computer. The monitoring content includes the stress, surface displacement and deformation damage characteristics of the roadway surrounding rock.

**Figure 4:**
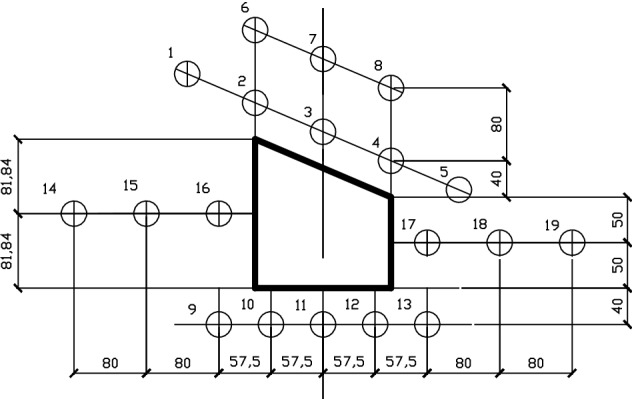
Layout of stress measuring points of roadway surrounding rock (mm).

**Figure 5:**
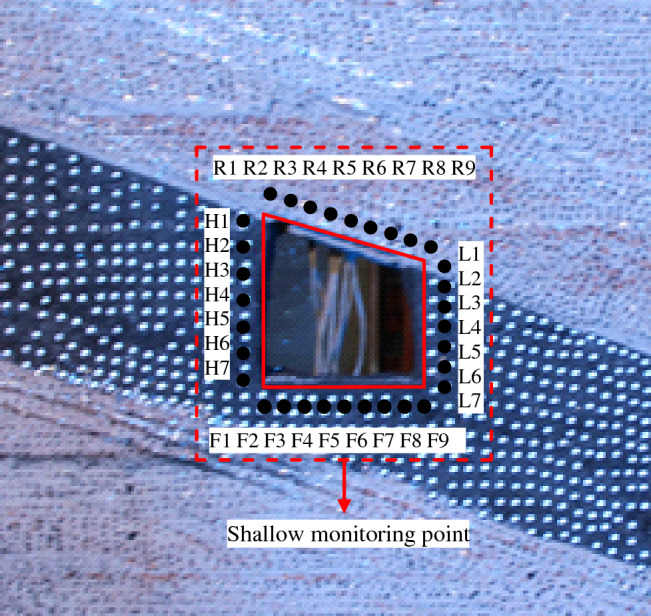
Layout of displacement measuring points of roadway surrounding rock.

**Figure 6:**
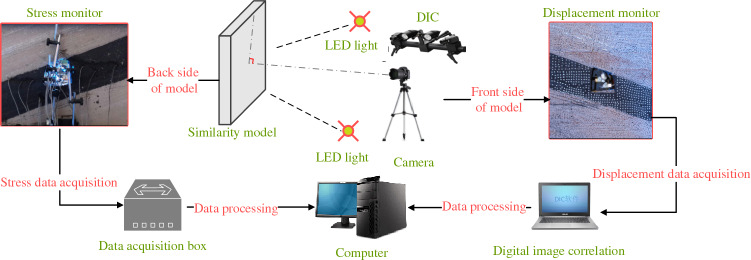
Flow chart of roadway deformation monitoring.

1. Stress monitoring of roadway surrounding rock
  According to the deformation and failure characteristics of inclined coal seam roadway in the actual project, and throughout the backfilling process, a total of 19 stress force sensors are embedded in the rock mass around the roadway (the roof, two sidewalls, floor and four sharp corners of the roadway) as schematically presented in Fig. 4 to measure the variation of rock stress during the roadway excavation and the surcharge loading process. The error of the stress sensor is 0.5% in the full range of measurement; whether the stress sensor fits closely with similar model materials and the installation method of the stress sensor may affect its measurement accuracy.
2. Roadway surface displacement monitoring
  In a similar physical simulation test, since most traditional measurement methods have low acquisition density, large discreteness, and low measurement efficiency, it results in a large measurement error^29^ (Chen et al. 2016). Therefore, this paper uses a test method to monitor the displacement and deformation failure characteristics of roadway surface—Digital Image Correlation (DIC), which has the advantages of non-contact, robustness, and full-field measurement and can overcome the weakness of traditional measurement methods^30^. DIC is introduced into the deformation measurement of a similar model, which has short monitoring time intervals and high precision. It can realize the high precision test of large range and detail parts of rock strata simultaneously, complete the continuous dynamic monitoring of similar material models, and obtain the deformation characteristics of the model in a small time scale. It provides rich monitoring information for the comprehensive study of the deformation and failure law of roadway surrounding rock to accurately grasp the dynamic process and local information of rock deformation, movement, failure, and collapse^31–35^. The resolution of the two CCD digital cameras is 2648 × 2448 pixels. Sub pixel accuracy can be achieved using 3D–DIC software (GOM-Aramis, version 8, related solutions). In this experiment, the photographing format is 1.2 × 1.1 m, and the magnification is 3.383 pixel / mm, and the accuracy of DIC displacement measurement is expected to be 0.0296 mm, and the layout of displacement monitoring points and DIC measurement system are shown in Figs. 5, 6. For geotechnical problems, the strain level measured by DIC is usually much higher than the typical measurement error range. The materials and scale dimensions of similar model tests are carefully designed. However, it is still difficult to avoid the development of plane strain, which may damage the plane strain conditions and affect the measurement results of DIC. At the same time, eight fixed control marks are placed on a similar model frame, and DIC can be used to eliminate the rigid body displacement to correct some measurement errors in the post-DIC treatment. Because the test object is a large plane similar model, the test focuses on the structural deformation of the model, focusing on the phenomena and laws presented in the test. Therefore, in this study, the use of DIC technology is helpful to measure the displacement and strain field around the roadway surrounding rock.

### Test procedures

The mixture of sand, calcium carbonate and plaster powder was mixed evenly with water, and then it was layered and filled into the model test apparatus to make the physical similarity model, in which the layered thickness was controlled at 1–3 cm. The stress sensor is embedded in the corresponding position. After the model is placed for one month, the overall moisture content of the model reaches 1.6–2.7%, and its similar materials reach the expected mechanical strength, round measuring points were pasted around the roadway and speckle patterns were prefabricated^36^. According to the size of the roadway, the sawing device is carefully used to expand the excavation range from the center of the roadway to the opening until the roadway contour is reached. Then the load is applied to the top of the model step by step until the roadway is completely destroyed. During the loading process, the DIC measurement system records the surface deformation of the model, and the stress sensor measures the stress of two sides, roof, floor and sharp corner of roadway^37–40^.

## Results and interpretation

### Displacement analysis of roadway

The displacement of the roadway surrounding rock directly reflects the deformation of surrounding rock under stress. Studying the displacement of different parts of roadway surrounding rock under different loads is an essential part of analyzing the roadway's deformation and failure mechanism.

#### Displacement of roadway surface

**Figure 7:**
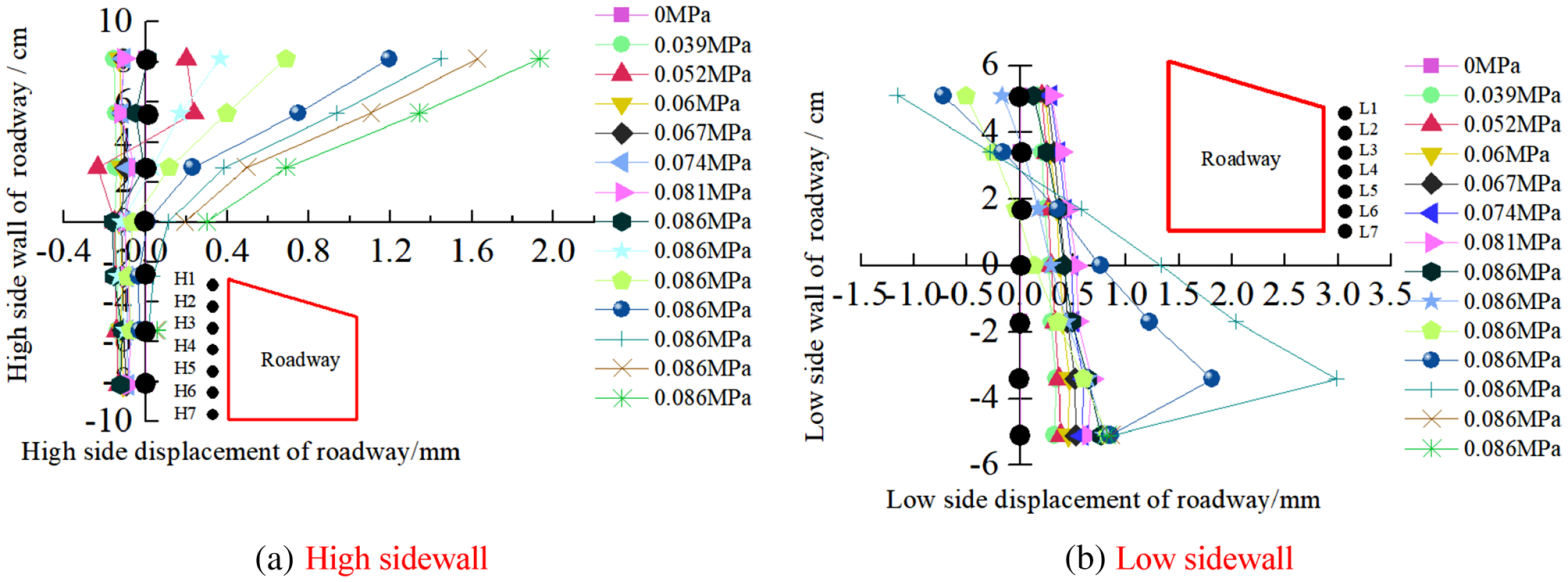
Evolution of surrounding rock on shallow surface of two sidewalls of roadway with loading.

**Figure 8:**
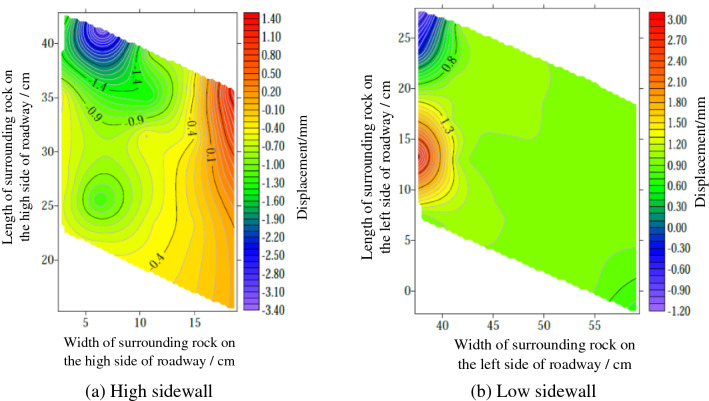
Displacement nephogram of all measuring points on both sides of roadway at 0.063 MPa.

**Figure 9:**
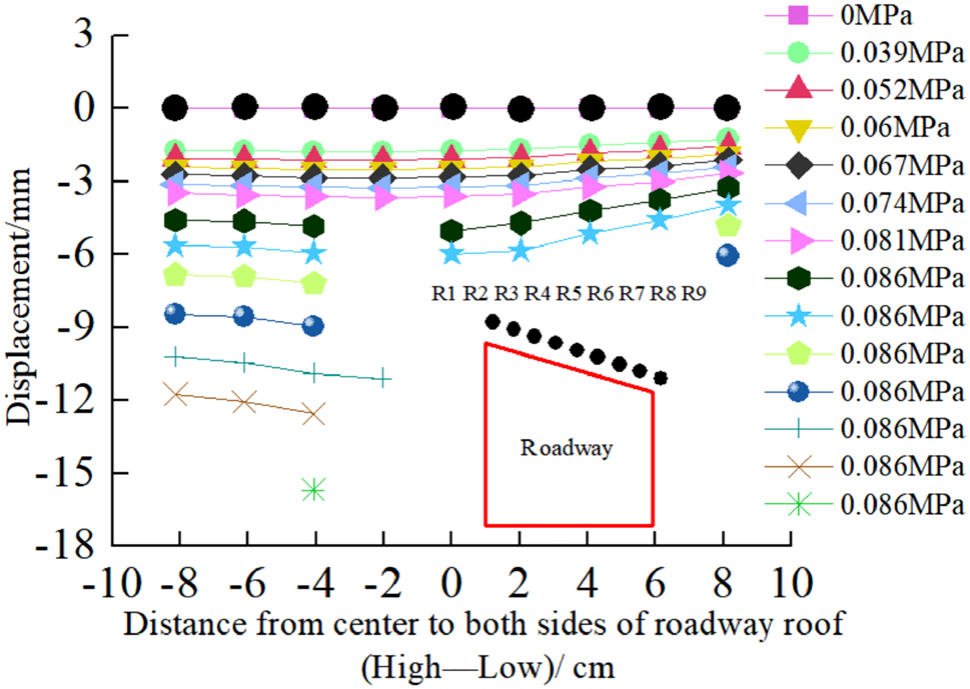
Evolution of surrounding rock separation layer on shallow surface of roadway roof with loading.

**Figure 10:**
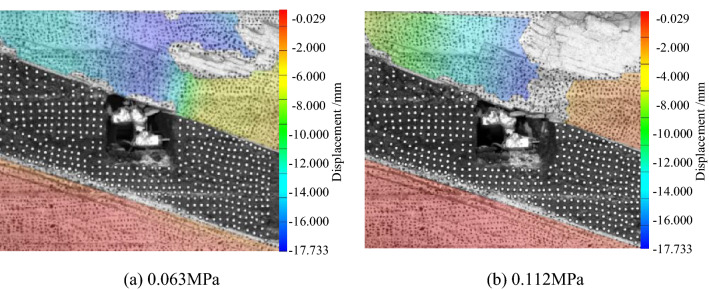
Displacement nephogram of all measuring points on roadway roof surface.

**Figure 11:**
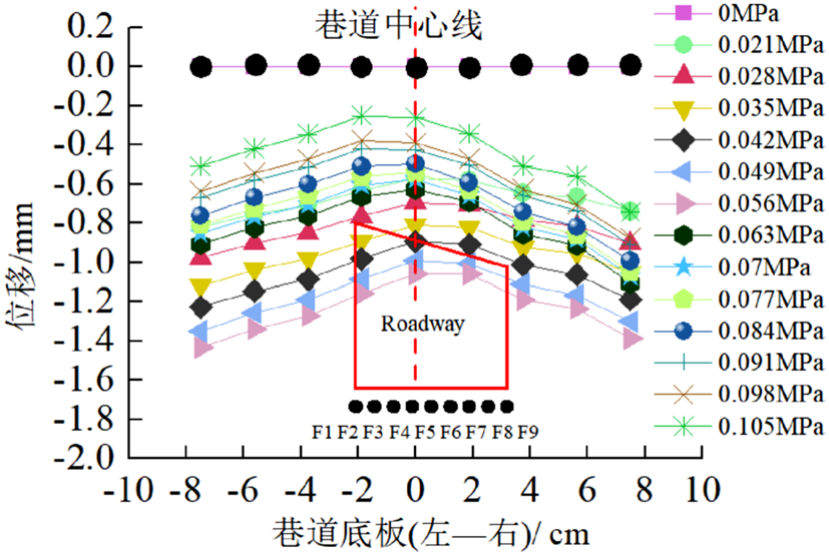
Evolution of floor heave of roadway with loading.

**Figure 12:**
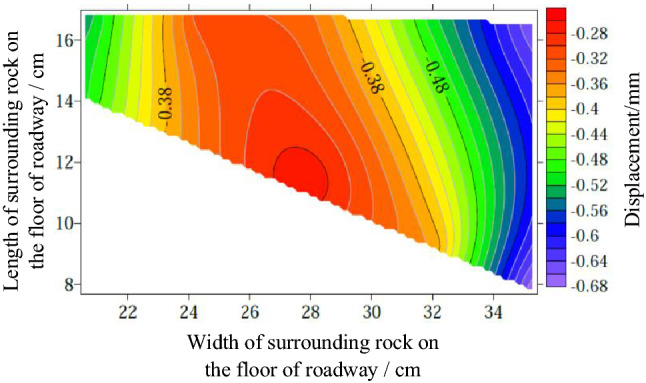
Displacement nephogram of all measuring points in roadway floor.

1. Surface displacement of two sidewalls of roadway
  Figure 7 shows the evolution of the surrounding rock on the shallow surface of the two sides of roadway with loading. It can be seen from the figure that under low load, the displacement of surrounding rock of the two sidewalls of roadway is small, and the surrounding rock of the two sidewalls of roadway is in the compaction stage. When loading to 0.063 MPa, the upper displacement of the two sidewalls of roadway began to increase, and the surrounding rock of the two sidewalls of roadway was bulging into the roadway. Combined with the displacement nephogram Fig. 8 of all the measured points on the two sidewalls of roadway, it can be seen that the deformation of the shallow surrounding rocks of the two sidewalls of roadway is large, and the upper side of the two sidewalls of the surrounding rocks severely bugles. With the increase of the distance from the surrounding rock in the roadway to the sidewall, the deformation is smaller, indicating that the deep surrounding rock of roadway is in the compaction stage and is relatively stable. With the continuous increase of load, the roadway's two sidewalls seriously bulge, and the maximum displacement of the high sidewall and the low sidewall is 1.934 mm and 2.98 mm, respectively. There is no data for the last loading of the low sidewall measuring point of the roadway, indicating that the surrounding rock of the roadway's low sidewall surface is spalling and the measuring point is damaged. As shown in Fig. 19d, the surface damage of the roadway's low sidewall is more significant than that of the high sidewall surface, which shows asymmetric characteristics.
2. Roof displacement of roadway
  Figure 9 is the evolution figure of the surrounding rock separation layer loading on the shallow surface of roadway roof. It can be seen from the figure that under the action of lower load, the displacement of each measuring point of roof has little difference, and the subsidence trend is similar, which indicates that the roadway roof has good integrity at this time. When the load reaches 0.063 MPa, the subsidence of each measuring point of roof increases sharply, especially the subsidence of the middle measuring point of roof is the largest, and its value is far greater than that of the other measuring points. With the continuous increase of the load, there is no data for the middle measuring point and the low sidewall measuring point of roof, indicating that the roof is separated from the layer and caving, and the deformation of the low sidewall and the middle surrounding rock of roof is large, which leads to the damage of the measuring point. At this time, the deformation of the middle and low sidewall of roadway roof is greater than that of the high sidewall, showing asymmetric characteristics, as shown in Fig. 19d. Combined with the displacement nephogram Fig. 10 of all measuring points on the roof surface of roadway, it can be seen that the deformation of surrounding rock in the middle of roadway roof is the largest at the early stage of loading. With the increase of load, the position of the maximum deformation of roadway roof is transferred from the high sidewall to the low sidewall, and the separation of surrounding rock of roadway roof begins to extend from the shallow part to the deep part. Finally, the deformation of the low sidewall and the low sidewall angle of roof is greater than that of the high sidewall.
3. Displacement of roadway floor
  Figure 11 is the evolution figure of floor heave with loading at shallow measuring points on the roadway floor's surface. It can be seen from the figure that under low load, the displacement of floor surrounding rock is small and is in the compaction stage. When the load is 0.063 MPa, the displacement of the surface measuring point on the low sidewall of the roadway floor is larger than that on the high sidewall, resulting in the subsidence of the low sidewall of roadway floor, the bulging of the high sidewall of floor, and the dislocation of the floor. As shown in Fig. 19a, b large crack appears on the floor's high sidewall, and a slight floor heave occurs on the roadway floor, shows asymmetric characteristics. Combined with the displacement nephogram Fig. 12 of all the measuring points in the floor, it can be seen that the deformation trend of the surrounding rock in the deep part of the roadway floor is consistent with that in the shallow part, and the larger the depth of the surrounding rock in the roadway floor is, the smaller the displacement is.

#### Internal displacement of roadway

**Figure 13:**
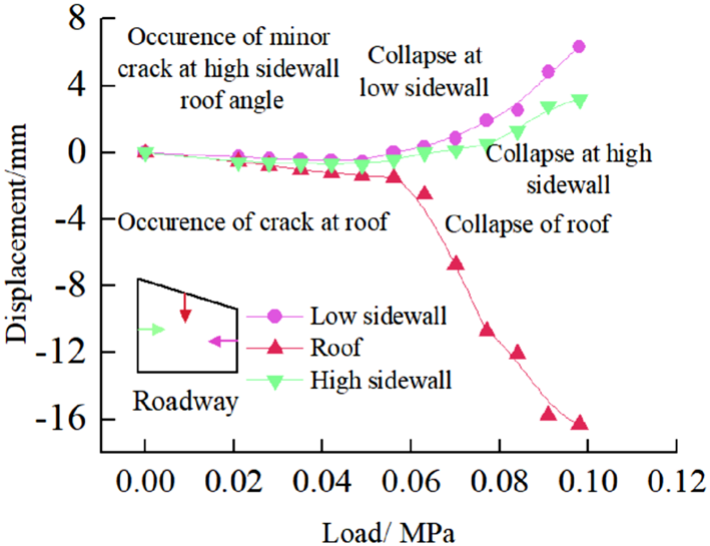
Variation of internal displacement of roadway with load.

The correlations between internal displacement of roadway and loading are plotted in Fig. 13. It can be seen that when the loading is at a low level (i.e. less than 0.063 MPa), both the internal displacement of roadway are insignificant. When the load increases beyond 0.063 MPa, the displacement at roof and two sidewalls increase rapidly, whereas the deformation of the two sidewalls of the roadway is more serious than that of the roof and the displacement of low sidewall is larger than that of high sidewall. The asymmetric displacement could be caused by dip angle of coal seam and section type of roadway.

### Strain analysis of roadway surface

**Figure 14:**
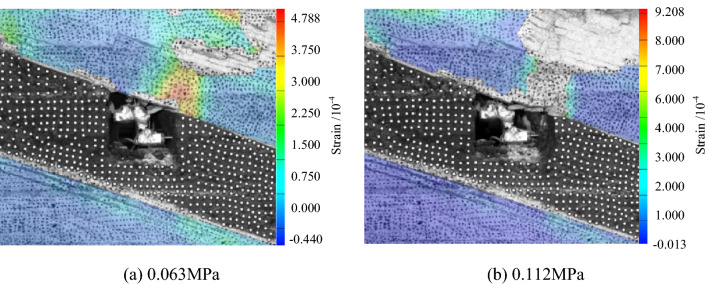
Strain nephogram of roadway surface.

Figure 14 shows the roadway's strain nephogram when the load reaches 0.07 MPa and the final failure surface of the roadway. It can be seen from the figure that when the load reaches 0.07 MPa, the strain of the roof of roadway is concentrated in the low sidewall, showing an asymmetric "Beret" type. Finally, the surface of the low sidewall of roadway roof is peeled off, the measuring point is invalid, and the asymmetric delamination collapse occurs. In this study, DIC technology explains the "Beret" of the surrounding rock above the roadway roof. The strain profile of type an asymmetric deformation reveals the process of asymmetric deformation and the failure of inclined strata.

### Stress analysis of roadway surrounding rock

The deformation and failure of the roadway in inclined coal seam are caused by the stress redistribution of surrounding rock. It is of great significance to study the stress distribution and change of surrounding rock under different loads to analyze the roadway's deformation and failure mechanism.

**Figure 15:**
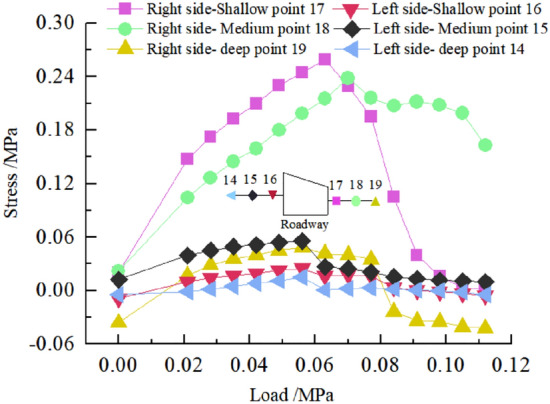
Curve of stress on two sides of roadway with load.

**Figure 16:**
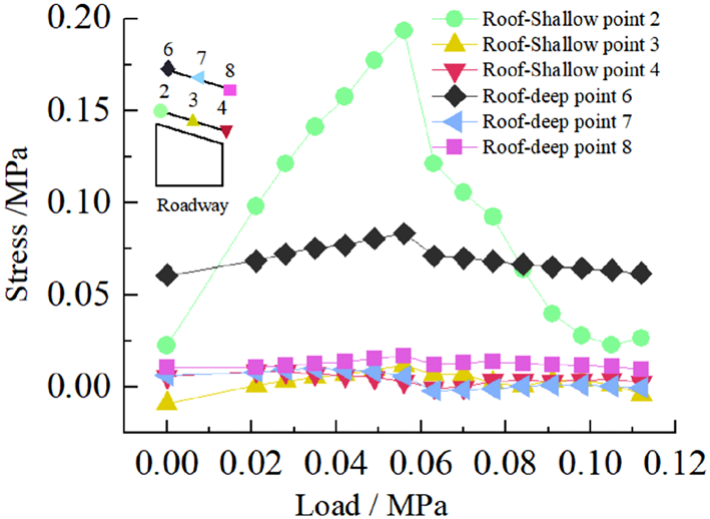
Curve of roadway roof stress with load.

**Figure 17:**
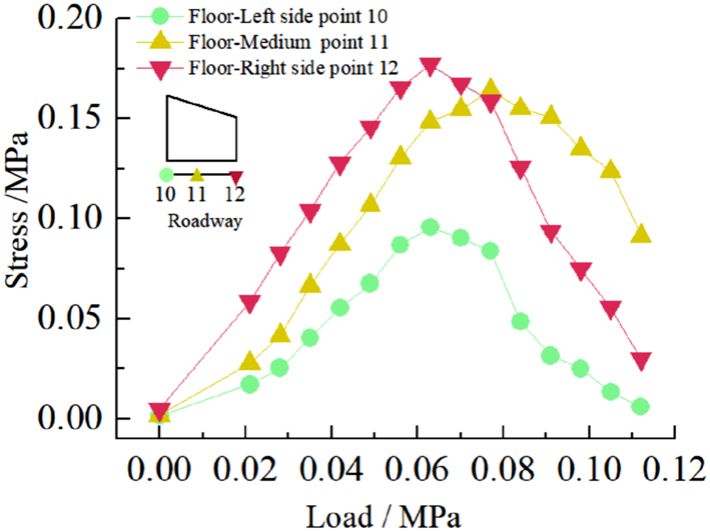
Curve of roadway floor stress with load.

**Figure 18:**
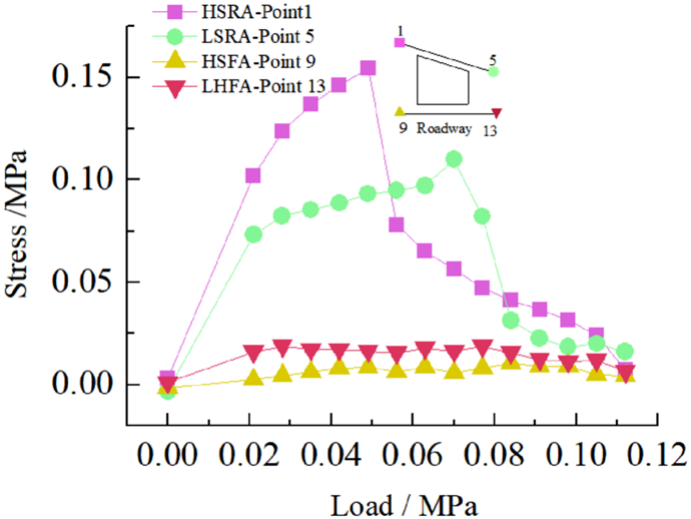
Curve of stress at sharp corner of roadway with load.

1. Stress distribution of two sidewalls of roadway
  Figure 15 shows the variation curve of surrounding rock stress of two sidewalls of roadway with loading. It can be seen from the figure that in the 0–0.063 MPa loading stage, the stress of 17 and 18 measuring points in the low sidewall of roadway increases rapidly, and the stress concentration occurs, while the stress of 19 measuring points in the low sidewall of roadway and all measuring points in the high sidewall of roadway increases steadily. When the load reaches 0.063 MPa, the stress concentration of roadway low-sidewall measuring point 17 reached the maximum, and the stress concentration coefficient was 4.1. The stress distribution of roadway high-sidewall measuring points 15 and 14 also reached the maximum, without stress concentration, and the stress value of high-sidewall measuring point 15 was the maximum. In the 0.063–0.112 MPa loading stage, the stress of 17 measuring points in the low sidewall of the roadway decreased sharply. Simultaneously, the stress of 18 measuring points in the roadway's low sidewall continued to increase to the maximum and then began to decrease, indicating that the stress concentration in the low sidewall of roadway transferred from the shallow to the deep. The stress of the high sidewall of roadway began to decrease slightly and finally stabilized. Overall, the stress of the low sidewall of roadway is greater than that of the high sidewall, showing asymmetric characteristics. The maximum stress of roadway's low sidewall appears in the shallow part of the roadway side, and the maximum stress of the high sidewall appears in the middle of the roadway side. The measuring point's stress value in the deep part of the two sidewalls of roadway is the smallest.
2. Stress distribution of roadway roof
  Figure 16 shows the variation curve of the surrounding rock stress of roadway roof with loading. It can be seen from the figure that in the early stage of loading, the stress of measuring point 2 of the roadway roof increases rapidly, and the stress concentration occurs. When the load reaches 0.056 MPa, the stress concentration of measuring point 2 rises to the maximum value, and the stress concentration coefficient is 3.4, while the stress of other measuring points of the roadway roof increases steadily. With the increase of load, the stress of each measuring point of the roadway roof began to decrease, and the stress of measuring point 2 of the roadway roof decreased the most. On the whole, the stress of surrounding rock at the high sidewall of roadway roof is the largest, followed by the stress at the low sidewall of roadway roof, and the stress at the middle of roadway roof is the smallest, which indicates that the stress concentration of roadway roof presents asymmetric characteristics.
3. Stress distribution of roadway floor
  Figure 17 shows the variation curve of surrounding rock stress of roadway floor with loading. It can be seen from the figure that under the action of load, the stress of each measuring point of roadway floor increases rapidly, and the stress concentration occurs. When the load reaches 0.063 MPa, the stress concentration of each measuring point in the roadway floor reaches the maximu, and the stress concentration factors of roadway floor low sidewall, middle and high sidewall measuring points are 2.8, 2.1 and 1.5, respectively. From this, it can be seen that the stress concentration of the low sidewall of floor is the largest, followed by the middle of roadway, and the minimum is the high sidewall of roadway, indicating that the stress distribution of the roadway floor is asymmetric.
4. Stress distribution at corner of roadway
  Figure 18 shows the variation curve of surrounding rock stress at the sharp angle of roadway with loading. It can be seen from the figure that the stress of the measuring point 1 of the high sidewall roof angle (HSRA) and the measuring point 5 of the low side roof angle (LSRA) of roadway increases rapidly with the increase of the load, and the stress concentration occurs. When the load is 0.049 MPa, the stress concentration of the measuring point 1 of the HSRA of roadway reaches the maximum value, the stress concentration coefficient is 3.1, and then begins to decline. The stress of the measuring point 5 of the LSRA of roadway continues to increase, and its stress concentration reaches the maximum value at 0.07 MPa, and the stress concentration coefficient is 1.57, indicating that the stress concentration of the HSRA of roadway transfers to the LSRA. The stress distribution at the high sidewall floor angle (HSFA) and low sidewall floor angle (LHFA) of roadway has small fluctuations, and the overall change is not large, indicating that the two sharp angles of floor have not been greatly damaged, and the surrounding rock is relatively stable. The size of the four sharp angles stress distribution value is: HSRA > LSRA > HSFA > LHFA.

### Deformation and failure characteristics of roadway surrounding rock

**Figure 19:**
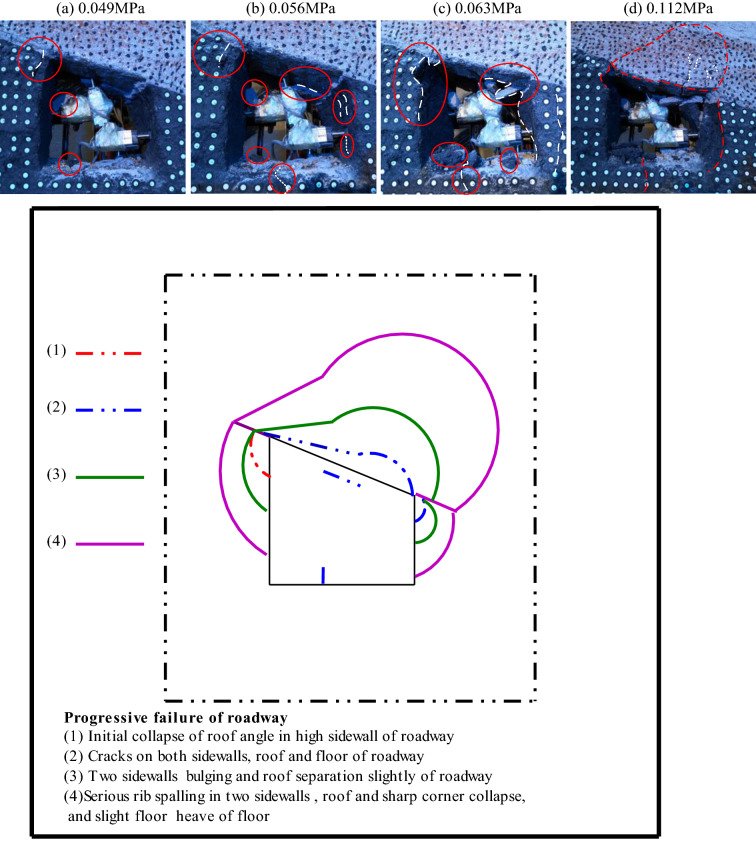
Cyclic failure of roadway.

The deformation and failure characteristics of the roadway surrounding rock are the most direct embodiment of its failure. With the increasing external load, the roadway's surrounding rock begins to deform slightly, and then cracks, crack propagation, local fracture, and overall failure occur, as shown in Fig. 19.

1. When the load reaches 0.049 MPa, the stress concentration of the roof angle of roadway's high sidewall reaches the maximum value, and the stress concentration coefficient is 3.1. The first fine cracks appear on the surface of the roof angle of the high sidewall of roadway. The fine cracks' length is 20–40 mm, and the roof angle of the high sidewall occurs local collapse, as shown in Fig. 19a.
2. When the load reaches 0.056 MPa, the stress of roof angle of the high sidewall of roadway begins to decrease, and the stress concentration shifts to the roof angle of the low side. The stress concentration on the roadway roof's high sidewall reaches the maximum value, and the stress concentration coefficient is 3.4. A large crack appears on the internal sidewall of roadway roof. The stress in the middle of roadway's high sidewall reaches the maximum, and there is no stress concentration. However, due to the influence of the roof sharp angles' stress concentration, there are fine cracks on the upper side of the roadway's high sidewall. The stress of the roadway's low sidewall and floor surrounding rock increases, and the stress concentration appears. The stress concentration of the roadway floor low sidewall is greater than that of the roadway's high sidewall. At this time, two large cracks appear in the roadway's low sidewall, and a crack appears in the roadway floor high sidewall, as shown in Fig. 19b.
3. When the load was 0.063 MPa, the roof angle of the roadway's high sidewall collapsed greatly, and the stress concentration at the roof angle of low sidewall of roadway reached the maximum value with the stress concentration coefficient of 1.57. The roof angle of the low sidewall of roadway collapsed locally. The stress of roadway roof and high sidewall began to decrease, the roadway roof separated from the layer, and a large main crack and many small cracks appeared in the high sidewall. The stress concentration of the shallow surrounding rock of the roadway's low sidewall reached the maximum value, with the stress concentration coefficient of 4.1. The roadway's low sidewall slightly bulged, and a crack appeared on the surface of the roadway's low sidewall. The stress concentration at the roadway floor's low sidewall reached the maximum value, with the stress concentration coefficient of 2.7, as shown in Fig. 19c.
4. When the load was 0.112 MPa, it is that the roadway's plastic deformation occurs at this time and the stress concentration of the surrounding rock of the low sidewall of roadway transfers to the deep surrounding rock of the low sidewall. Continuous loading until the roadway is completely destroyed, the two angles of roadway roof collapse and the failure of roof angle of the low sidewall of roadway is greater than that of the high sidewall. The roof of roadway is separated and collapsed, showing an asymmetric ''Beret'' type arch failure, and the cracks in the roadway floor deepened, and slight floor heave occurred, but the stress at the two corners of the roadway floor changed little and was relatively stable. Finally, only one crack appeared at the low sidewall angle of floor surface. The two sidewalls of roadway are seriously fragmented. The upper side of the roadway's high sidewall is partially collapsed, and the low sidewall of roadway is bulging, all of which are collapsed. The surrounding rock of roadway surface is exfoliated, and the damage of the roadway's low sidewall is greater than that of the high sidewall, showing asymmetric characteristics, as shown in Fig. 19d.

### Cyclic deformation mechanism of roadway surrounding rock

Under the action of load, the failure of the right angle trapezoidal roadway in inclined coal seam originates from the sharp corner of the roadway roof, and then the roadway roof, floor, and two sides begin to damage. The interaction of various areas of roadway surrounding rock leads to the increase of stress and the decrease of strength. The roadway surrounding rock enters a vicious cycle of deformation until the roadway presents the characteristics of asymmetric failure. Since the section type of inclined coal seam roadway is a right-angle trapezoid, the stress concentration is easy to occur at the sharp angle of the roadway roof, and the stress concentration at the sharp angle of the high sidewall of the roof first reaches the maximum value, resulting in cracks. With the increase of load, the roof's stress concentration, two sidewalls, and roadway floor also reached the maximum. At this time, cracks appeared in the roof's internal sidewall, the high sidewall of the floor and the two sidewalls, and then the roof was slightly separated, the two sidewalls bulged, and the floor of roadway was slightly heaved. As the load continues to increase, the stress concentration of the roadway's low sidewall transfers to the deep, and the failure of the surrounding rock of the low sidewall of the roadway extends from the shallow to the deep. At this time, the two sidewalls of roadway appear spalling and slight collapse, and the failure of the low sidewall of roadway is greater than that of the high sidewall, which leads to the decrease of the support capacity of roadway, increases the span of roadway, and aggravates the separation of the roof. The deformation of the roof increases the pressure of the two sidewalls and intensifies the two sides' failure. As the connection part, the corner continues to deteriorate the stress state. Finally, the two sidewalls' failure extends to the deep part, which deteriorates the roof's stress conditions. The roof rapidly collapses from the floor, showing an asymmetric "Beret" type of caving arch failure. That is, the roadway has two sidewalls, two sharp angles of the roof, and the roof falls into a vicious cycle that increases each other's stress, weakens the material, and intensifies the failure. This cycle continues until the roadway is completely unstable.

It can be seen from the above that the two sidewalls, roof and roof sharp angle of roadway are the key positions of roadway deformation and failure and show asymmetric characteristics. Therefore, the key to breaking the vicious circle and controlling the roadway's stability is to improve the asymmetric stress state of the roadway surrounding rock, adopt the supportive measures of asymmetric and key parts strengthening and improve the strength of roadway surrounding rock.

## Support design

### Principle of support design

According to the principle of cyclic failure and asymmetric failure of right angle trapezoidal roadway in inclined coal seam, the control principle of surrounding rock deformation is proposed:

1. In order to increase the support strength of roadway surrounding rock and improve the asymmetric stress state of roadway surrounding rock. The roof of roadway is supported by anchor net, anchor bolt and anchor cable combined with asymmetric support. Because the roof of roadway is destroyed by asymmetric ''Beret'' type caving arch, the roof anchor bolt and anchor cable are arranged by inclined installation and inclined to the low sidewall. The two sidewalls of the roadway are asymmetric supported by anchor net and anchor bolt. The stress concentration and deformation failure of the roadway's low sidewall is greater than those of the high sidewall, so the anchor bolt installation density should be appropriately increased in the same area.
2. Strengthening the support of weak parts of roadway. The deformation of the two corners of the roadway roof is serious, and the anchor bolt and anchor cable should be set up. Anchor bolt and anchor cables with sharp angles of the high sidewall tend to the high sidewall, and anchor cables with sharp angles of the low sidewall tend to the low sidewall, so as to strengthen the control of high-stress concentration and deformation and failure at the corner. It is also necessary to strengthen the support of the local position of the bottom boundary. By reducing the risk of damage at both ends of the floor, the instability of the whole floor is prevented, and the bearing capacity of the roadway is improved.
3. The supporting parameters of the anchor bolt and anchor cable are optimized to reinforce the surrounding rock of roadway. The length of the anchor bolt and anchor cable is determined by calculating the range of loose circle of roadway roof and two sidewalls, and the anchor cable should be anchored in the stable rock layer in the deep of roadway roof to ensure that the anchor cable can provide stable and long-term suspension force.
4. In order to control the stability of the surrounding rock on the surface of the roadway, the metal mesh should be used to support the two sidewalls of roadway.

### Parameters of support design

**Figure 20:**
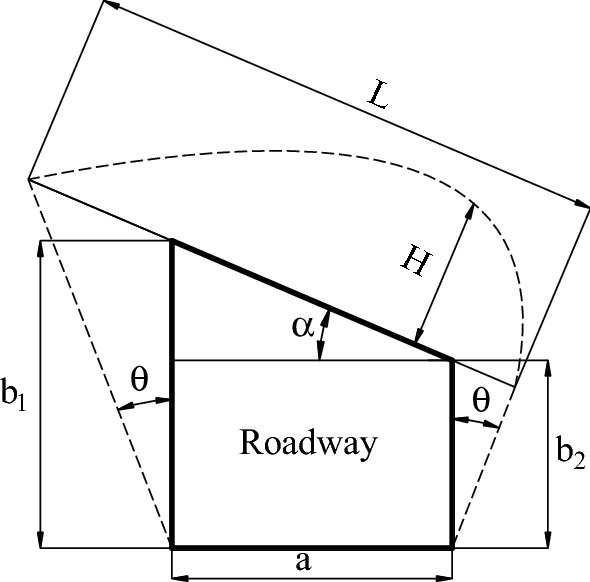
Loose ring of roadway roof.

According to the deformation control principle of asymmetric roadway in inclined coal seam, considering the asymmetric characteristics of surrounding rock stress, deformation and failure and the range of loose circle, combined with the support scheme of inclined coal seam roadway in No. 2 mining area of shitanjing, the asymmetric support design was carried out, and the combined support of anchor bolt, anchor cable and metal mesh was adopted. The theoretical calculation was carried out according to Pu's theory and elastic mechanics. The specific support parameters are as follows:

Due to the influence of dip angle on the roof of inclined coal seam roadway, the stress distribution of the two sidewalls shows asymmetric characteristics, which leads to the asymmetric "Beret" type caving arch of the roof loose circle of the roadway (as shown in Fig. 20). According to Pu's theory, the range of roof loose circle *H* is shown in Eq. (2):

2 $$ H = \frac{L}{f}\cos \alpha = \frac{{a\cos \varphi + \left( {b_{1} + b_{2} } \right)\tan \left( {45^{ \circ } - \frac{\varphi }{2}} \right)}}{2f} $$

where *H* is the range of roof loose circle; *α* is the dip angle of rock stratum, is 23°; *a* is the width of roadway, and its value is 3 m; *b*_*1*_ the height of high sidewall of roadway, and its value is 4.91 m; *b*_*2*_ is the height of low side of roadway, and its value is 3 m; *f* is the Pu's coefficient of roof rock, and its value is 2.3; *φ* is the internal friction angle of the rock mass in the two sidewalls, and its value is 28°; *c* is cohesion, and its value is1.4; The calculation results show that the range of roadway roof loose circle is 1.93 m.

Then the length *L*_*R*_ of roof anchor bolt is shown in Eq. (3):

3 $$ L_{R} = L_{R1} + H + L_{R2} $$

where *L*_*R*_ is the length of roof anchor bolt; *L*_*R1*_ is the exposed length of roof anchor bolt, and its value is 0.15 m; *L*_*R2*_ is the anchorage length of roof anchor bolt, and its value is 0.55 m; *H* is the range of roof loose circle, and its value is 1.93 m;

According to engineering analogy and experience, the length of roof anchor bolt is 2.7 m, and the row spacing of roof anchor bolt is 0.8 m.

**Figure 21:**
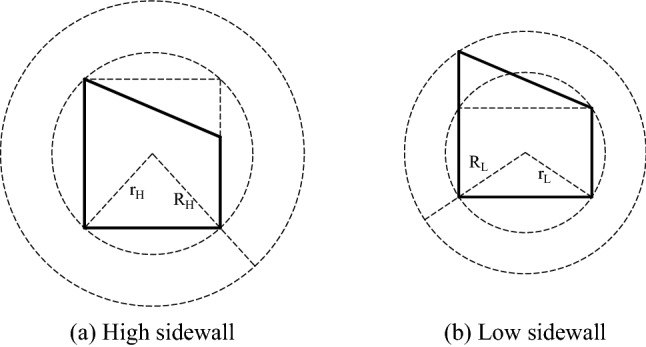
Loose circle of two sidewalls of roadway.

Due to the plastic zone of the two sidewalls of the right angle trapezoidal roadway in inclined coal seam is asymmetric, the section form is simplified to different circular roadways (as shown in Fig. 21). According to the elastic–plastic mechanics, the range of the loose circle of the high sidewall and the low sidewall of the roadway is *R*_*H*_ and *R*_*L*_, respectively, as shown in Eqs. (4) and (5):

Where *R*_*H*_ and *R*_*L*_ are the range of loose circle of high and low sidewalls of roadway, respectively; *P* is vertical original rock stress, and its value is 10 MPa; *α* is the dip angle of rock stratum, and its value is 23°; *a* is the width of roadway, and its value is 4.5 m; *b*_*1*_ the height of high sidewall of roadway, and its value is 4.91 m; *b*_*2*_ is the height of low side of roadway, and its value is 3 m; *φ* is the internal friction angle of the rock mass in the two sidewalls, and its value is 28°; *c* is cohesion, and its value is 1.4. The calculation results show that the range of the left side loose circle is 1.58 m, and the range of the right side loose circle is 1.1 m.

The length of the high and low sidewall anchor bolts of the roadway is shown in (4) and (5), respectively:

4 $$ L_{HS} = L_{S1} + R_{H} + L_{S2} $$

5 $$ L_{LS} = L_{S1} + R_{L} + L_{S2} $$

where *L*_*LS*_ is the length of low-sidewall anchor bolt; *L*_*S1*_ is the exposed length of two sidewalls anchor bolt, and its value is 0.15 m; *L*_*S2*_ is the anchorage length of anchor bolts, and its value is 0.45 m;

According to the engineering analogy and experience, the length of the left side anchor bolt is 2.2 m, the length of the right side anchor bolt is 2.0 m, and the row distance between the two sides of the roadway is 0.8 m.

**Figure 22:**
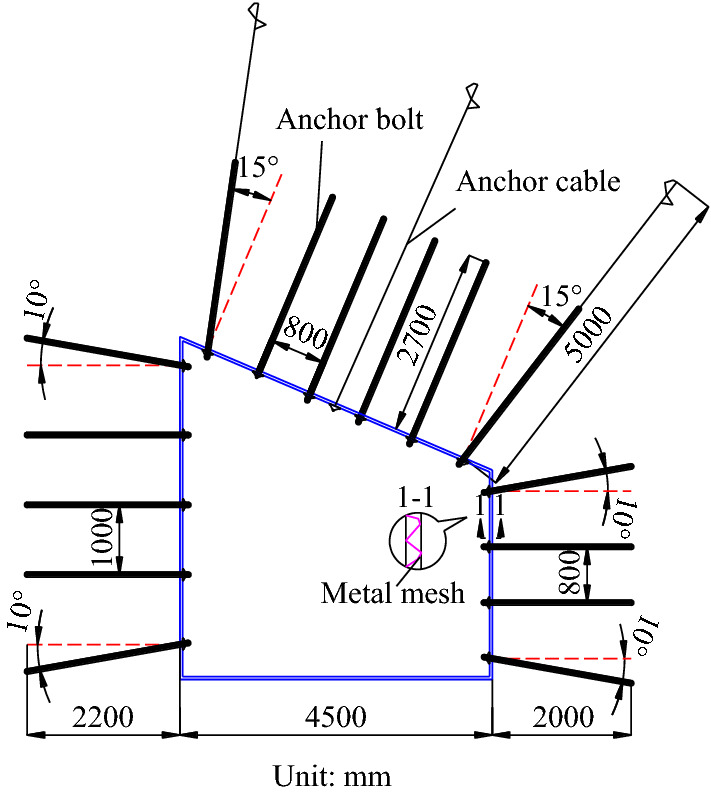
Support scheme of roadway.

According to the above theory's calculation results and the asymmetric deformation and failure of the roadway and the existing support experience, and a new support scheme was developed for the roadway of No.2 Mining Srea of Shitanjing, as shown in Fig. 22. The following support systems were applied:

1. *Support pattern* Combined support system with anchor bolt, anchor cable, beam, and metal mesh.
2. *Roof support* Threaded steel anchor bolts with a diameter of 20 mm and length of 2700 mm were used for full-length anchoring. The spacing between the bolts (arranged in a row) was 800 mm × 800 mm. Steel strands with a diameter of 15.24 mm and length of 5000 mm were used as anchor cables, and the spacing between the cables (arranged in a row) was 2400 mm × 2400 mm. I-shaped steel with length of 1200 mm and aperture of 16 mm were used as supporting beams of anchor cable. The anchor cables were connected by #14 channel steel with a length of 2400 mm, the steel ladder beam is 4.0 m in length and 50 mm in width, and the metal mesh was installed with a specification of 2.0 m × 1.0 m.
3. *Sidewall support* Threaded steel anchor bolts with a diameter of 20 mm, length of high sidewall 2200, and length of low sidewall 2000 mm were used for full-length anchoring. According to the principle of asymmetric support, the low side is encrypted. The spacing between the bolts of high sidewall and low sidewall (arranged in a row) was 1000 mm × 800 mm, 800 mm × 800 mm, respectively. The same parameters as the roof support scheme were used for the metal mesh and steel ladder beams.
4. *Local enhancement support* One threaded steel anchor bolt was installed to four sharp corners of roadway at an angle of 10° with respect to the horizontal direction.

## Support effect of the roadway

### Support effect of numerical simulation

#### Establishment of model

**Figure 23:**
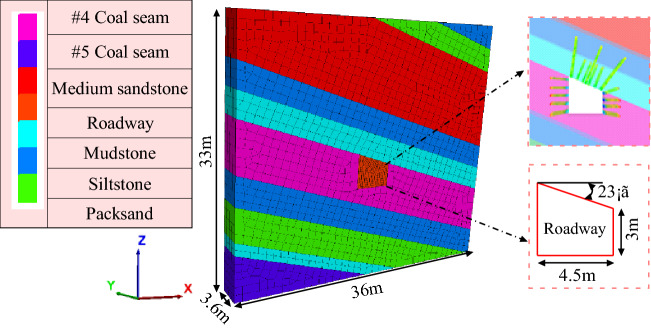
3D computing model.

Based on the engineering geological conditions of Shitanjing No. 2 mine, the numerical calculation model is established by using FLAC3D software. The model size is 33 m × 3.6 m × 36 m, the dip angle of the coal seam is 23°, and 10 MPa vertical stress is applied on the top surface of the model to simulate the action of in-situ stress. Considering the calculation accuracy, the mesh around the roadway is densified, and the model is divided into 13,381 units, as shown in Fig. 23. After the roadway is excavated, the roadway is supported according to the design parameters. Physical and mechanical parameters and support material parameters are shown in Tables 3 and 4 respectively.

**Table 3:** Numerical simulation of physical and mechanical parameters of coal and rock.

Rock stratum	Thickness (m)	Density (g cm^3^)	Bulk modulus (GPa)	Shear modulus (GPa)	Friction (°)	Cohesion (MPa)	Tension (MPa)
#3 Coal seam	3.15	1.40	2.00	1.10	26.0	1.20	1.00
Siltstone	3.00	2.46	8.49	6.47	32.1	5.7	3.77
Packsand	3.00	2.5	8.24	5.92	30.16	9.62	2.27
Medium Sandstone	10.00	2.51	10.11	7.27	37.0	11.8	2.78
Siltstone	3.00	2.46	8.49	6.47	32.1	5.7	3.77
Mudstone	3.00	2.53	7.79	5.34	31.5	1.85	1.54
#4 Coal seam	6.00	1.40	2.00	1.10	28.0	1.40	1.00
Siltstone	3.50	2.46	8.49	6.47	32.1	5.7	3.77
Packsand	4.00	2.5	8.24	5.92	30.16	9.62	2.27
Mudstone	1.50	2.53	7.79	5.34	31.5	1.85	1.54
#5 Coal seam	5.5	1.40	2.00	1.10	26.0	1.20	1.00

**Table 4:** Support material parameters.

Support material	Elastic modulus (GPa)	Tension (MPa)	Cross sectional (area/m^2^)	Adhesive force of anchoring agent (MPa)	Anchorage agent stiffness (N/m)
Roof anchor bolt	200	455	0.00125		
Two sides anchor bolt	210	370	0.000803		
Anchor bolt anchorage section				3.2	6 × 10^8^
Anchor cable	195	1860	0.000706		
Anchor cable anchorage section				3.2	6 × 10^8^

#### Result analysis

**Figure 24:**
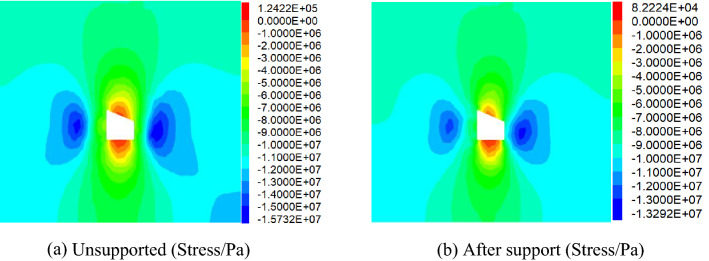
Vertical stress nephogram of the roadway.

1. Comparative analysis of vertical stress and horizontal stress of roadway
  Figure 24, 25 shows the vertical and horizontal stress nephogram of before and after roadway support. It can be seen from the figure that after roadway support, compared with that without support, the vertical stress distribution of roadway surrounding rock is uniform, which makes the original skewed stress asymmetric distribution transfer to the middle of roadway roof and floor, and the difference of stress concentration distribution between the two sides of roadway is significantly reduced. The horizontal stress concentration range, numerical value and difference at each sharp corner of the roadway are reduced. It shows that under the action of asymmetric support, the asymmetric stress distribution of roadway is significantly improved, the asymmetric problem of roadway stress distribution is effectively solved, and the stability of roadway is well controlled.
2. Comparative analysis of vertical stress and horizontal displacement of roadway
  Figure 26, 27 shows the vertical and horizontal displacement nephogram of before and after roadway support. It can be seen from the figure that after roadway support, the maximum subsidence of roadway roof is reduced from 127.3 to 44.0 mm, the maximum floor heave of roadway is reduced from 73.0 to 28.2 mm, the maximum displacement of right side of roadway is reduced from 130.0 to 33.8 mm. and the maximum displacement of left side of roadway is reduced from 110.0 to 31.1 mm. It shows that the deformation of roof, floor and two sides before and after roadway support is significantly reduced, and the asymmetric deformation of roadway is effectively controlled.
3. Comparative analysis of plastic zone of roadway surrounding rock
  Figure 28 Plastic zone nephogram of roadway surrounding rock. It can be seen from the figure that after roadway excavation, under the action of stress redistribution, the distribution of plastic zone of roadway surrounding rock presents asymmetric characteristics, that is, the range of plastic zone on the right side of roadway is larger than that on the left side, and the roadway surrounding rock presents shear failure. After roadway surrounding rock support, the distribution range of plastic zone decreases, and the plastic zone on the left and right sides is more uniform. It shows the improvement of roadway surrounding rock deformation.

**Figure 25:**
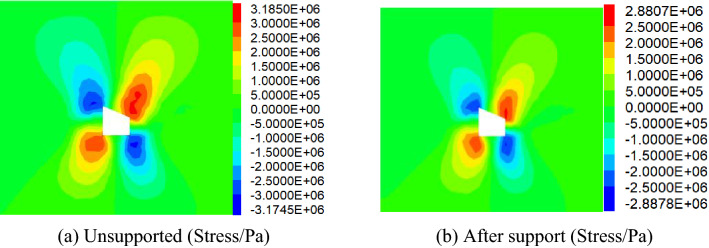
Horizontal stress nephogram of the roadway.

**Figure 26:**
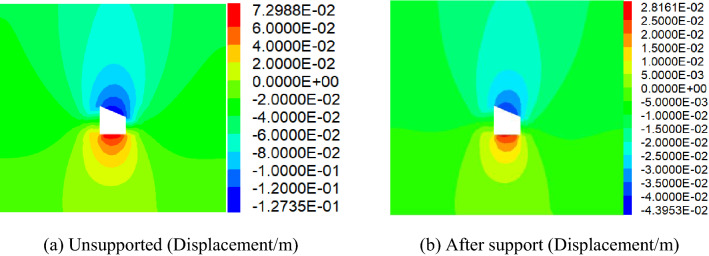
Vertical Displacement nephogram of the roadway.

**Figure 27:**
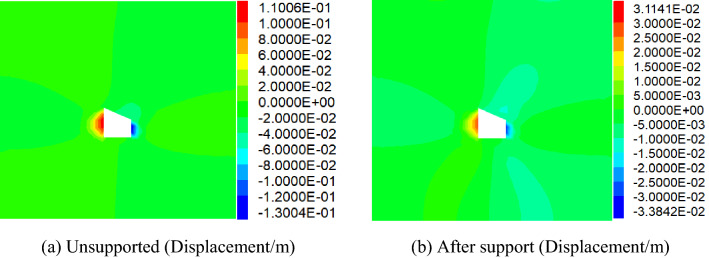
Horizontal Displacement nephogram of the roadway.

**Figure 28:**
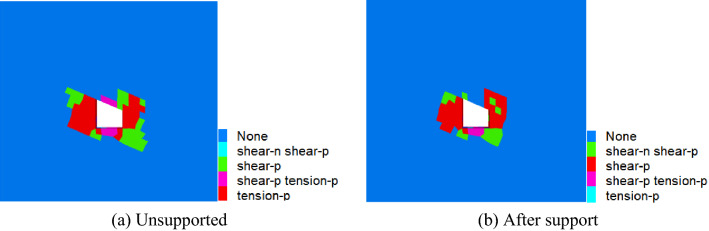
Plastic zone nephogram of the roadway.

### Field measured support effect

**Figure 29:**
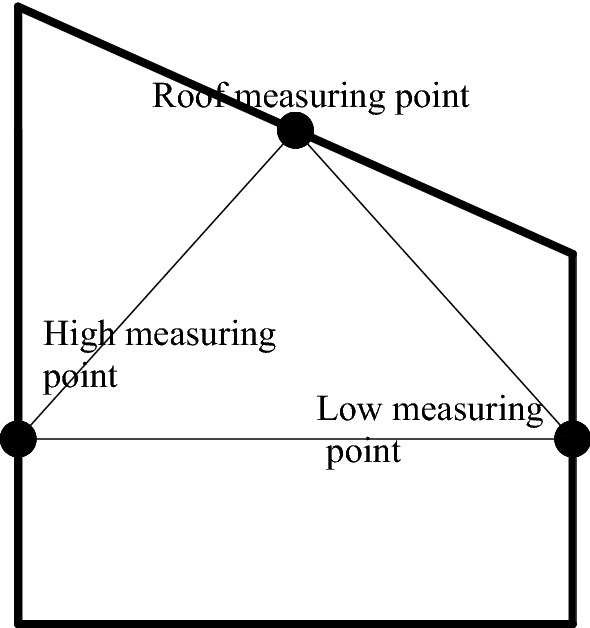
Layout of roadway surface displacement monitoring points.

**Figure 30:**
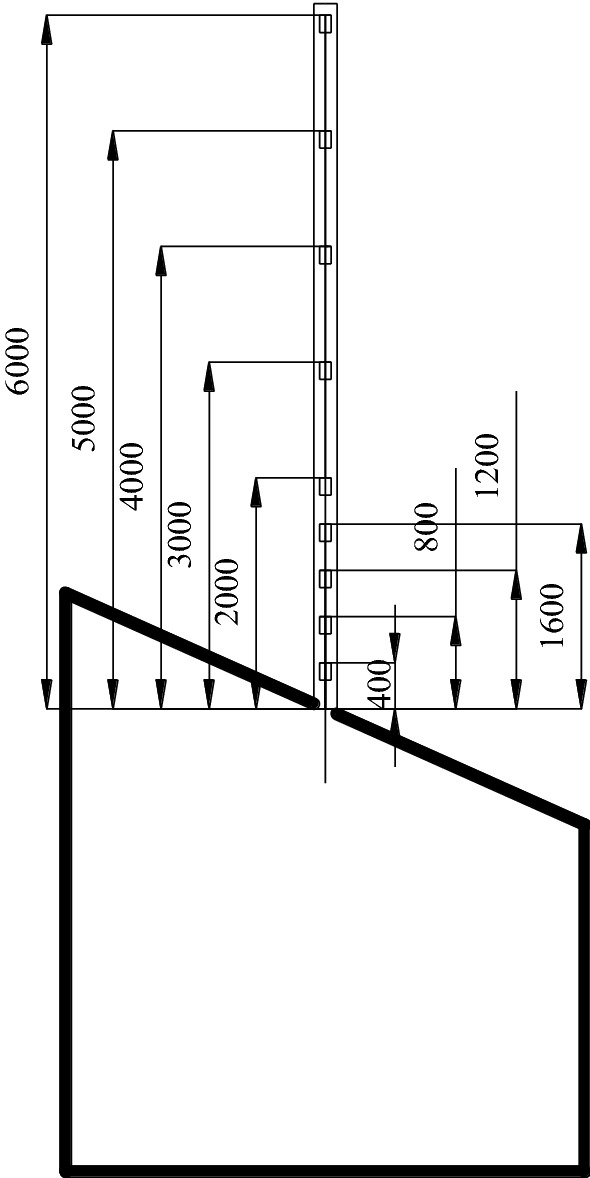
Layout of roadway roof separation monitoring points (mm).

**Figure 31:**
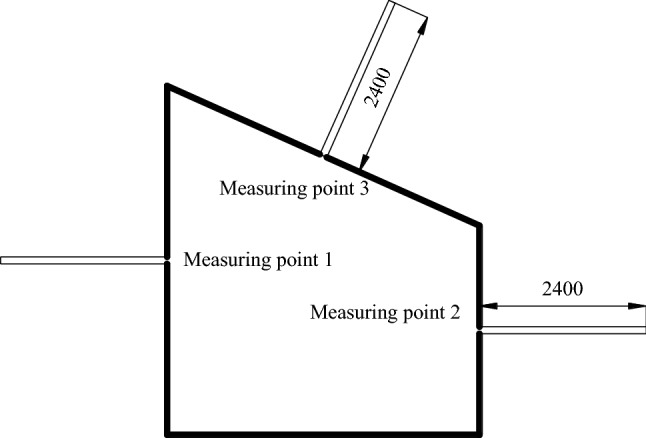
Layout of monitoring points of roadway loose circle (mm).

**Figure 32:**
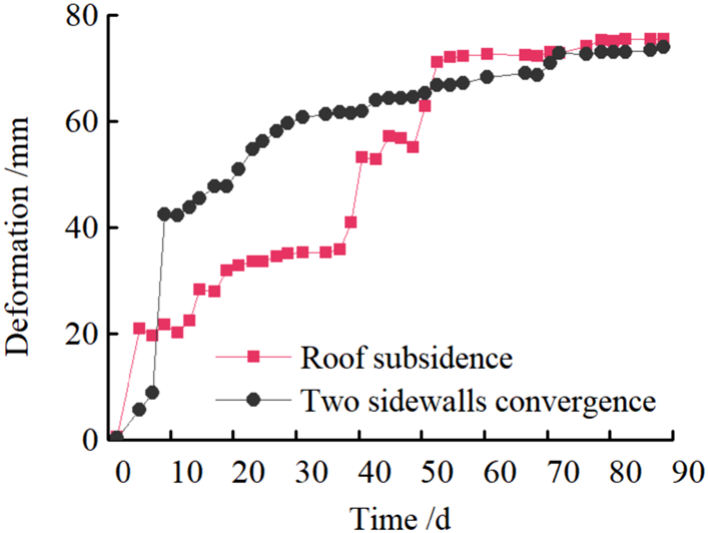
Deformation of Roadway.

**Figure 33:**
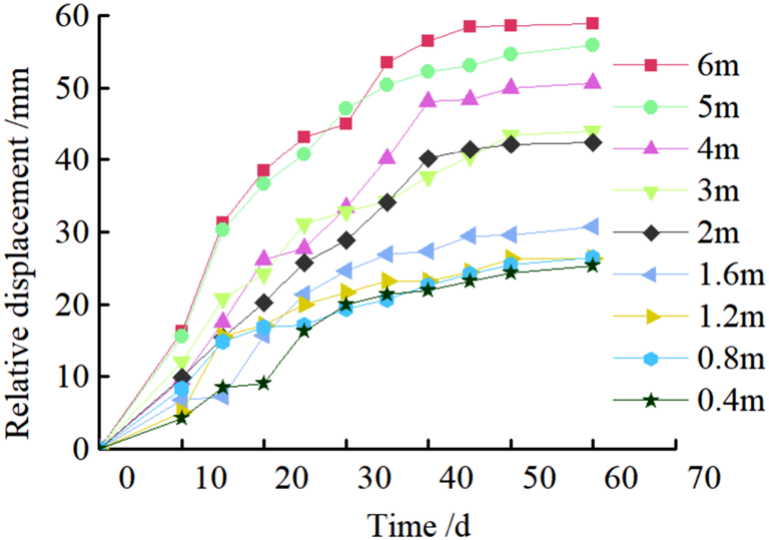
Roof Separation.

**Figure 34:**
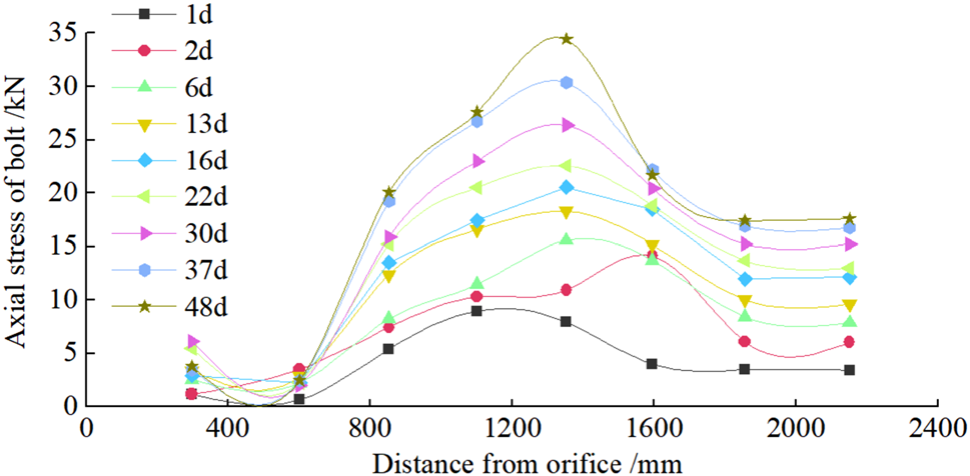
Axial Force of Anchor Bolt.

**Figure 35:**
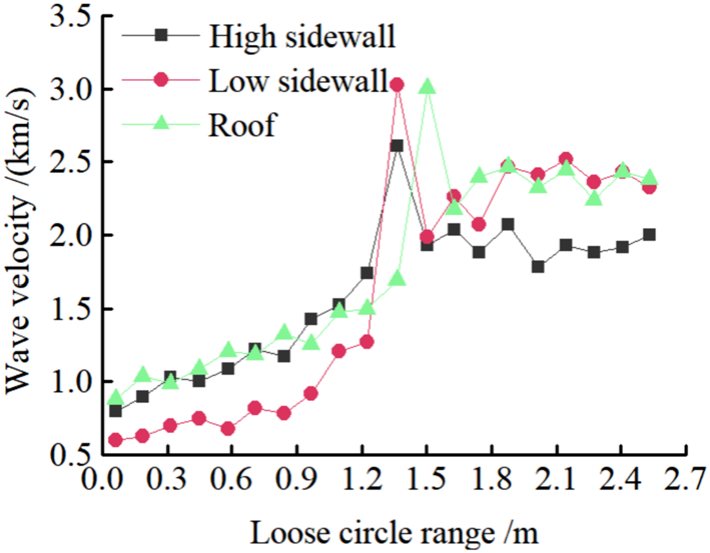
Loose circle test curve.

In order to detect the effect of asymmetric roadway support, the support scheme is applied to the mining roadway of the working face, and the monitoring station is arranged in the typical section of the surrounding rock of the roadway, and the layout of monitoring points is shown in Figs. 29, 30, and 31. The monitoring results are shown in Figs. 32, 33, 34, and 35.

It can be seen from Figs. 32, 33, 34, and 35, the maximum settlement of the roof is 75 mm, and basically stable after 13 days. The maximum relative convergence of the two sidewalls is 74 mm, which is basically stable after 15 days. The maximum roof separation occurred in 1.2–1.6 m. After 12 days, the roof separation stabilized, and the maximum separation was 10 mm, and its maximum separation occurred in the range of anchor bolt support. The maximum axial force of the roof anchor bolt is 35 kN. The loose circle range of the roadway roof is 1.4–1.7 m, and the loose circle range of two sidewalls is 1.2–1.5 m, which is basically consistent with the results of theoretical calculation. The subsidence of the roof, the convergence of the two sidewalls, and the stress of the anchor bolt are less than the design requirements, indicating that the asymmetric support scheme effectively controls the deformation of the surrounding rock of the roadway, improves the stability of the roadway, and the support effect is good. It can be seen that the asymmetric support technology of inclined coal seam roadway has good applicability.

## Conclusion

In this paper, the stress distribution law and the cyclic failure mechanism of the right-angle trapezoidal roadway in inclined coal seam are investigated systematically through a series of physical model tests with the facilitation of non-contact full-field displacement measurements using the DIC technology. The asymmetric support measures are proposed, verified by numerical simulation and applied to engineering practice. The following conclusions are drawn:

1. It is found that the stress distribution of surrounding rock of right angle trapezoidal roadway in inclined coal seam is asymmetric, and the maximum stress concentration coefficient of two sidewalls, roof and floor are 4.1, 3.4 and 2.8, respectively. The stress concentration of the low sidewall of the roadway is significantly greater than that of the high sidewall, and the distance from the stress concentration position of the low sidewall to the sidewall of the roadway is greater than that of the high sidewall. With the increase of load, the stress concentration of the two sidewalls transfers to the deep. The stress concentration of the high sidewall of roadway roof is greater than that of the low sidewall in the early stage of loading. With the increase of load, the stress concentration of high sidewall transfers to low sidewall. The stress concentration of the low sidewall of the roadway floor is greater than that of the high sidewall. The size of the four corner stress distribution value is: HSRA > LSRA > HSFA > LHFA.
2. The concept of "cyclic deformation failure" of right angle trapezoidal roadway in inclined coal seam is put forward, that is, the roadway's failure originates from the sharp angle of roof and sidewall of roadway, and the cyclic interaction of the two sidewalls, the sharp angles and roof aggravated the failure of roadway, resulting in the overall instability of roadway. Due to the influence of asymmetric stress concentration of roadway surrounding rock, its deformation and failure also show asymmetric characteristics. The sidewall of the roadway is most seriously damaged, and the low sidewall is greater than the high sidewall. Roof separate layer caving, showing asymmetric "Beret" type caving arch failure. The corner deformation and failure of the low sidewall of the roof are greater than that of the high sidewall. There is a large crack at the high sidewall of the bottom plate and a slight floor heave. There is no large damage at the two corners of the bottom plate and only the crack at the corner of the low sidewall.
3. Based on the principle of cyclic failure and asymmetric failure of right angle trapezoidal roadway in inclined coal seam, some asymmetric support principle of roadway surrounding rock in inclined coal seam was proposed. In brief, the principles aimed to increase the support strength to the right angle trapezoidal roadway in inclined coal seam on the whole, optimize the support parameters of anchor bolts and anchor cables through the theoretical calculation of roadway surrounding rock loose circle, enhance the support to vulnerable regions in the right angle trapezoidal roadway in inclined coal seam, and improve the support to the roof and sidewalls to control deformation and failure of roadway. The roof anchor bolt and anchor cable are arranged by inclined installation, and the low-sidewall anchor bolts are densely supported, and the bolt support is added at four sharp corners.
4. Numerical analysis is carried out before and after the support of FLAC3D inclined coal roadway. After the roadway support, the asymmetric stress distribution characteristics of surrounding rock are significantly improved, and the deformation of surrounding rock of roadway is effectively controlled. Among them, the maximum subsidence of roadway roof is reduced from 127.3 to 44.0 mm, the maximum floor heave of roadway is reduced from 73.0 to 28.2 mm, the maximum displacement of right side of roadway is reduced from 130.0 to 33.8 mm. and the maximum displacement of left side of roadway is reduced from 110.0 to 31.1 mm.
5. The asymmetric support scheme was later verified by on-site engineering practice. Under this scheme, the maximum value of roof subsidence was 75 mm, the maximum cumulative deformation value of two side walls was 74 mm, The maximum roof separation occurred in 1.2–1.6 m, and the maximum separation was 10 mm. The maximum axial force of the roof anchor bolt is 35 kN. The loose circle range of the roadway roof is 1.4–1.7 m, and the loose circle range of two sidewalls is 1.2–1.5 m. Thus, the asymmetric support scheme successfully met the requirements for safe mining operations.
